# A Comparative In Vitro Analysis of the Osteogenic Potential of Human Dental Pulp Stem Cells Using Various Differentiation Conditions

**DOI:** 10.3390/ijms21072280

**Published:** 2020-03-26

**Authors:** Terezia Okajcekova, Jan Strnadel, Michal Pokusa, Romana Zahumenska, Maria Janickova, Erika Halasova, Henrieta Skovierova

**Affiliations:** 1 Department of Medical Biochemistry, Jessenius Faculty of Medicine in Martin, Comenius University in Bratislava, 03601 Martin, Slovakia; okajcekova1@uniba.sk (T.O.); romana.zahumenska@gmail.com (R.Z.); 2 Biomedical Center Martin, Jessenius Faculty of Medicine in Martin, Comenius University in Bratislava, 03601 Martin, Slovakia; jan.strnadel@uniba.sk (J.S.); michal.pokusa@uniba.sk (M.P.); henrieta.skovierova@uniba.sk (H.S.); 3 Department of Stomatology and Maxillofacial Surgery, University Hospital in Martin, 03601 Martin, Slovakia; maria.janickova@uniba.sk; 4 Department of Medical Biology, Jessenius Faculty of Medicine in Martin, Comenius University in Bratislava, 03601 Martin, Slovakia

**Keywords:** dental pulp stem cells, differentiation, osteogenesis, regenerative medicine

## Abstract

Dental pulp stem cells (DPSCs) have excellent proliferative properties, mineralization potential and can be easily obtained from third molar teeth. Recently, many studies have focused on isolation and differentiation of DPSCs. In our study, we focused on biological properties of non-differentiated DPSCs in comparison with osteogenic differentiated cells from DPSCs. We analyzed morphology as well as mineralization potential using three varied osteogenic differentiation media. After fifteen days of differentiation, calcium deposit production was observed in all three osteogenic differentiation media. However, only one osteogenic medium, without animal serum supplement, showed rapid and strong calcification—OsteoMAX-XF™ Differentiation Medium. Therefore, we examined specific surface markers, and gene and protein expression of cells differentiated in this osteogenic medium, and compared them to non-differentiated DPSCs. We proved a decrease in expression of CD9 and CD90 mesenchymal stem cell surface markers, as well as downregulation in the expression of pluripotency genes (*NANOG* and *OCT-4*) and increased levels of expression in osteogenic genes (*ALP, BSP, OCN* and *RUNX2*). Moreover, osteogenic proteins, such as BSP and OCN, were only produced in differentiated cells. Our findings confirm that carefully selected differentiation conditions for stem cells are essential for their translation into future clinical applications.

## 1. Introduction

Despite breakthroughs in medicine and medical technology, this industry will still face considerable challenges which will only increase in difficulty throughout the 21st century. An exponentially growing global population, coupled with an increase in the amount of elderly, are increasing the needs in the health care sector worldwide. Nowadays, research into stem cell regenerative properties in bone healing has increased. Bone disorders are often connected with old age, low physical activity and overweightness or obesity. Bone surgery can be helpful in the case of small defects, but larger defects are correlated with more complications, such as dehiscence of bone during secondary infections, and pathological fractures. Studies focused on the replacement of damaged tissue by allogenic or autologous transplantation show some limitations [1,2]. Moreover, there are still open questions regarding the osteogenic differentiation level of stem cells, e.g., application of non-differentiated or differentiated cells, the fate of stem cells inside the bone, or the response to the treatment. 

Dental pulp is a soft connective tissue that is not mineralized. The main function of dental pulp is to produce dentin and to maintain the physiological vitality of the tooth. Outside of the pulp tissue is a layer of odontoblast cells, which are responsible for the secretion and mineralization of the extracellular matrix of dentin [3]. In case of delicate stimulus, odontoblasts produce increased levels of reactionary dentin [4]. Contrarily, in the case of firm stimulus or dead odontoblasts, new odontoblasts are necessary to regenerate dentin and to protect the vitality of dental pulp. These odontoblasts are differentiated from the stem cells found in pulp tissue [5,6]. Dental pulp stem cells (DPSCs) are an attractive option for regenerative medicine and tissue engineering because they can be easily isolated from third molar teeth extracted for orthodontic or clinical reasons. DPSCs can be expanded and can generate the huge number of cells necessary to cover grafted materials [7]. Moreover, their high mineralization potential can be useful in bone regeneration therapy [8]. 

DPSCs have mesenchymal stem cell characteristics, which include their capacity for self-renewal and multipotent differentiation that is not only typical for mesodermal cell lineages, like osteogenic, chondrogenic and adipogenic lineages, but also for ectodermal and endodermal cell lineages, such as active neurons, hepatocyte-like cells, cardiomyocytes, melanocytes and myocytes [5,9]. However, there are some disadvantages associated with DPSC application. The quality and quantity of mesenchymal stem cells are the main factors for obtaining optimal outcomes of tissue regeneration [10]. The culture conditions can positively or negatively affect the properties of stem cells. In contrast to bone marrow-derived mesenchymal stem cells (BMMSCs), DPSCs are reported to maintain an undifferentiated state even during long-term cultivation and they have an increased number of passages as well as higher capability for osteogenic differentiation [11,12]. 

DPSCs and their differentiation potential from the perspective of dental regenerative medicine are a main focus of our research group. Therefore, in the present study, we have concentrated on the biological properties of DPSCs and analyzed their osteogenic potential using three differentiation media to determine the best one for future use in clinical application. The composition of these media is the confidential property of each company. However, we conducted the comparative analysis of osteogenic properties of DPSCs using xeno-free differentiation media by biological, molecular–biological and cytological methods. OsteoMAX-XF^TM^ Differentiation Medium (Medium C) showed the best osteogenic-differentiated properties in comparison to the other two osteogenic-differentiated media.

## 2. Results

### 2.1. Osteogenic Differentiation of DPSCs

**Figure 1:**
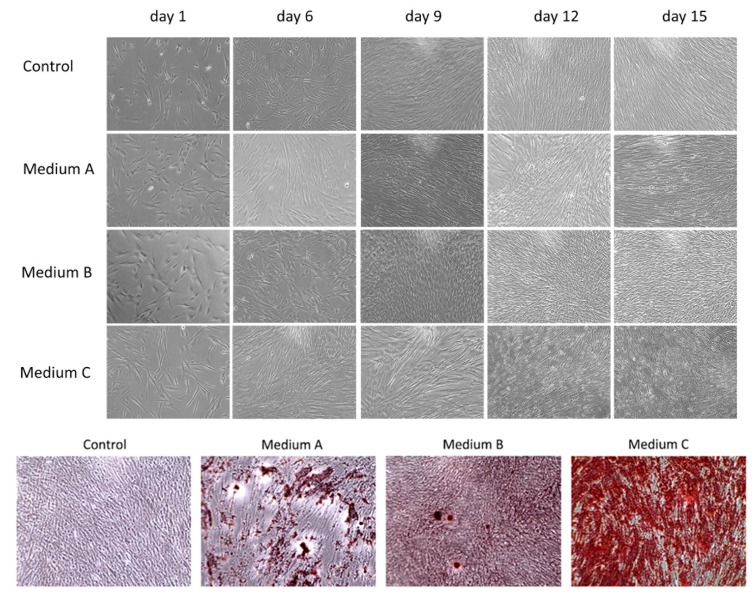
Morphological changes during osteogenic differentiation of dental pulp stem cells (DPSCs) by phase-contrast microscopy. Three differentiation media ((**A**)–(**C**)) were used to monitor cell morphology and proliferation as well as calcium deposit production for fifteen days. Control group was cultured in basic growth media. After fifteen days of differentiation, calcium-rich deposits were stained via Alizarin Red S. Magnification 100×.

In this study, we compared the osteogenic properties of DPSCs using various differentiation media. Initially, DPSCs as mesenchymal stem cells showed a spindle-shape or fibroblast-like morphology with adherence to the surface and colony formation ability growing in a swirl-like pattern (Figure 1). The profile of typical CD markers expression for DPSC as Mesenchymal stem cells is presented in Supplementary Figure S1. After six days of differentiation, cells cultured in Medium C (OsteoMAX-XF™) became elongated when compared to other cultures and had an orderly arrangement with a tendency to align themselves into parallel lines. On the ninth day of cultivation, cells in both Medium C and Medium A (StemPro^®^) stopped their proliferation and started to produce calcium deposits. The amount of these deposits increased during prolonged differentiation (up to fifteen days) in all three osteogenic differentiation media, but in Medium C (OsteoMAX-XF™) it was most obvious. In addition, the changes in cell morphology were most noticeable in Medium C in comparison to the control group (Figure 1). 

In order to detect calcium deposits, which are indicators of differentiated osteoblasts, the cell cultures were fixed and stained with 2% Alizarin Red S. The amount and intensity of red-colored calcium compounds were monitored by light microscopy (Figure 1). No mineralized nodules were observed in the control sample.

### 2.2. Flow Cytometry Analysis

**Figure 2:**
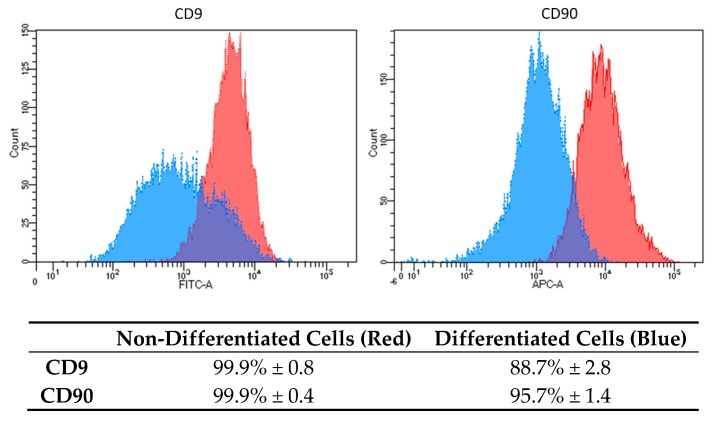
Surface markers expression characteristics in DPSCs differentiated into osteoblasts. Histograms show differences in the expression level of two specific CD markers (CD9 and CD90) in non-differentiated cells (red color) and in differentiated cells (blue color). Results are presented as mean percentage (%) of positivity in the total population ± standard deviation (SD).

Based on the results, we decided to analyze the osteogenic properties of DPSC cells induced by OsteoMAX-XF™ Differentiation Medium (Medium C). To confirm the mesenchymal stem cell’s phenotype, flow cytometry analysis was performed on non-differentiated and differentiated DPSCs cells using two fluorescently labelled monoclonal antibodies against surface markers CD9 and CD90. Analysis was performed on 20,000 events and gating was set up based on an unstained/isotype control (Figure 2).

### 2.3. Comparative Analysis of Gene Expression for Stem and Osteogenic Markers

**Figure 3:**
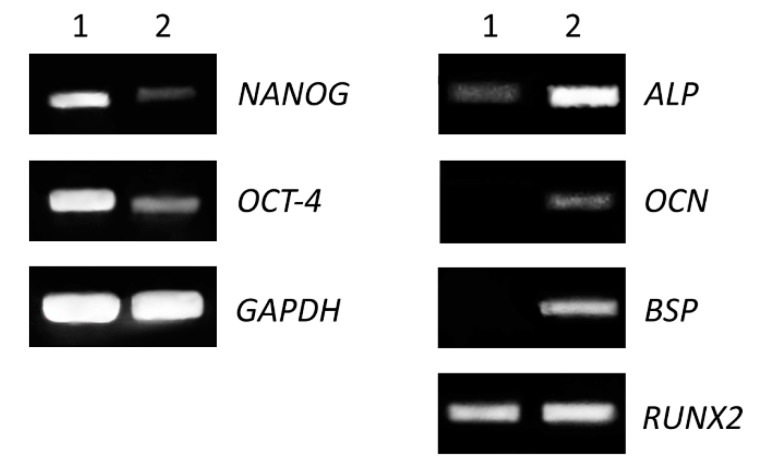
Comparison of expression level of stem cells and osteogenic markers. Non-differentiated (1) and differentiated (2) cells were seeded in 25 cm^2^ flasks for 15 days. Total RNA was isolated and expression of pluripotent genes (**left panel**), transcriptional factor (*NANOG*) and octamer-binding transcription factor 4 (*OCT-4*), as well as osteogenic genes (**right panel**) alkaline phosphatase (*ALP*), bone sialoprotein (*BSP*), osteocalcin (*OCN*) and runt-related transcription factor 2 (*RUNX2*) were analyzed by RT-PCR. The housekeeping gene glyceraldehyde 3-phosphate dehydrogenase (*GAPDH*) was used as an internal control.

The expression of genes specific for mesenchymal stem cells and for osteogenic-differentiated cells (Medium C) was performed on cells grown in 25 cm^2^ flasks as the monolayer for fifteen days. We detected decreased levels of gene expression for stem cell transcriptional factors (*NANOG, OCT-4*) and increased expression of genes encoding proteins involved in osteogenesis (alkaline phosphatase, bone sialoprotein, osteocalcin and runt-related transcription factor 2) (Figure 3). 

### 2.4. Immunocytochemistry of Osteogenic Proteins

**Figure 4:**
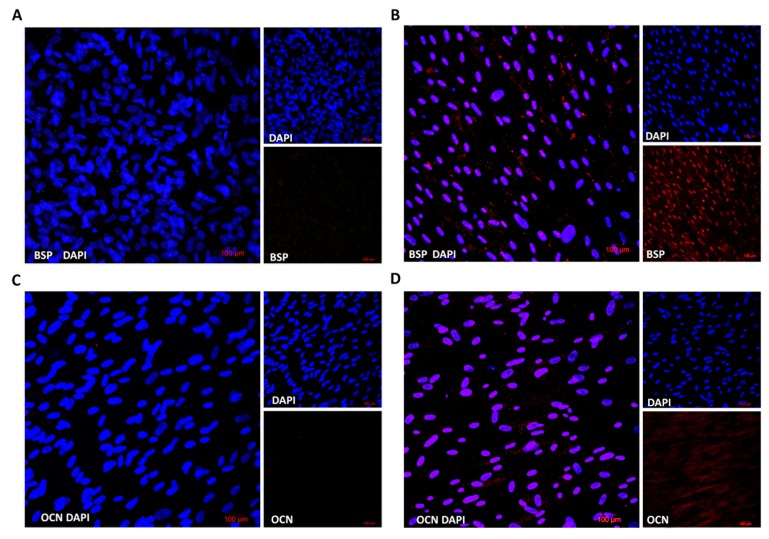
Immunofluorescence analysis of specific osteogenic markers. Non-differentiated (**A**,**C**) and osteogenic differentiated cells (**B**,**D**) were fixed and stained by antibodies BSP or OCN, respectively. Nuclei were visualized by DAPI. Stained cells were analyzed by confocal microscopy.

We also evaluated the protein expression of osteogenic markers in differentiated cells in comparison to stem cells using multiphoton confocal microscopy. Cells were grown on microscopy slides and were differentiated into osteoblasts for fifteen days. Fluorescent microscopy showed that differentiated cells expressed both osteogenic markers, bone sialoprotein (BSP) as well as osteocalcin (OCN), which distinguished them from stem cells (Figure 4). These results proved the gene expression profile where the expression of the *BSP* gene was stronger than *OCN*. 

## 3. Discussion

Recently, stem cell therapy and tissue engineering have been commonly considered as a great approaches for the treatment of various diseases. Autologous and allogenic bone transplantation is one of the gold standard treatments in bone defect reparation which could be caused by various diseases, infections, congenital defects or trauma [13,14]. Moreover, bone grafting techniques have several limitations and disadvantages, including post-operative pain, infection risk, donor side morbidity, as well as immunological problems, and the search for suitable bone substituents that will mimic the osteogenic potential of autologous bone is needed [15]. Recently, several studies have been focused on mesenchymal stem cell differentiation potential in bone regeneration, while avoiding invasive surgical procedures because they have the potential to renew themselves for long periods through cell division and, under certain physiological or experimental conditions, they can be induced to become specialized cells [16,17].

In this study, we focused on the osteogenic potential of human dental pulp stem cells, which can be translated into clinical application in the future. Therefore, we decided to compare three different osteogenic media to find the best one for future basic research or clinical application using various scaffolds which could be applied in tissue engineering. The composition of differentiation media is confidential. However, one of the factors which can dramatically affect osteogenic differentiation is the presence of animal serum [18,19]. Fetal serum has been routinely used in osteogenic mesenchymal stem cell cultures, but its xenogeneic origin could have two potential disadvantages in future clinical applications: (i) the problem of adverse immune reactions and (ii) the problem with xenogeneic infections. Consequently, the application of animal-derived cell culture supplements could contaminate cells with xenoantigens [20]. Based on our knowledge, only one differentiation media had a xeno-free composition: Medium C (OsteoMAX-XF^TM^ Differentiation Medium). Cells differentiated with this medium showed the changes in cell morphology a short time after osteoinduction (six days) when compared to control. Cells became elongated and started to change from stem cells to progenitor osteoblast cells, as they started to reduce proliferation and to induce cell differentiation. This was noticed by calcium deposit production after nine days of osteoinduction. When we counted the cells after fifteen days of differentiation, we observed significantly lower numbers of differentiated cells (1 × 10^6^ cells) compared to control cells (3 × 10^6^ cells), which also points out cellular and metabolic changes. DPSCs in the other two differentiation media, Medium A (StemPro^®^ Osteogenesis Differentiation Kit) and Medium B (Mesenchymal Stem Cell Osteogenic Differentiation Medium) also started to produce calcium deposits, but they took a longer time (more than twelve days) and their intensity of deposit production was weaker, especially in the case of Medium B. 

The visualization of calcium phosphates and carbonates, which are indicators of functional osteoblasts, was conducted using Alizarin Red S staining. We observed mineralized nodules in all three osteoinductive media (Figure 1). However, the calcium deposit production varied in each of them. The same results, as observed with a morphological comparison of the cytological changes, were proven even when using deposit staining (Figure 1). Medium C showed the strongest calcium mineral production. In addition, we observed some calcium production in Medium A. On the other hand, only a few mineral calcium nodules were stained when Medium B was used. 

Based on our results, we decided to use only Medium C to analyze other biological properties of osteogenic-differentiated cells compared to DPSCs. CD9 is a transmembrane protein and a representative surface marker for DPSCs stemness [21]. We found that CD9 expression varied in other types of cells used in our lab. Based on our experimental knowledge, we decided to use CD9 as one of the markers, which can distinguish between dental stem cells and other types of cells (Skovierova, unpublished data) [21]. CD 90 is a GPI-anchored glycoprotein 1 and is one of the typical markers for mesenchymal stem cells [17,22,23]. Both surface markers were highly expressed in non-differentiated DPSCs, 99.9% (Figure 2, red-colored peaks). Moreover, the expression of CD9 and CD90 was homogenous and strong. After differentiation, we analyzed the expression and distribution of both CD markers (Figure 2, blue-colored peaks). In the case of CD9, an evident decrease in intensity (2.6-fold reduction in mean fluorescence intensity and reduction of positive cells from 99.9% to 88.7%) was found. As for CD90, the changes were strong (9.6-fold reduction in mean fluorescence intensity and reduction of positive cells from 99.9% to 95.7%). This indicates dramatic changes in the biological properties of DPSCs and their stemness reduction.

The osteogenic differentiation of DPSCs requires the activity of specific transcription factors, which bind to the DNA of stem cells and regulate their gene expression during bone formation [24]. These transcription factors are expressed and functional at distinct time points during differentiation, which refer to various developmental stages of the osteoblast lineage [23,25]. We proved that the expression of *alkaline phosphatase (ALP), bone sialoprotein (BSP)* and *osteocalcin (OCN*) genes increased during the differentiation of DPSCs (Figure 3, right panel). Moreover, increased protein expression was analyzed by fluorescent microscopy, and both proteins (BSP and OCN) were detected only in osteogenic-differentiated cells compared to control (Figure 4). 

Transcription factor RUNX2 is one of the main regulators of osteogenic differentiation. It acts throughout the induction, proliferation and maturation of osteoblasts and regulates expression of many other genes involved in this process [26,27]. In addition, the expression of both genes, *BSP* and *OCN,* is regulated by *RUNX2*. We noticed similar levels of the expression of *RUNX2* in differentiated as well as in non-differentiated cells. This could be due to longer cultivation of non-differentiated cells. DPSC cells were highly overgrown in control after fifteen days of culture and they could have started to differentiate spontaneously. The high confluence could be one of the factors of increased expression of *RUNX2* in the control. On the other hand, gene expression of stem cell-associated markers (*NANOG*, *OCT-4*) was evidently decreased in differentiated cells (Figure 3, left panel). NANOG is a transcription factor in embryonic stem cells and is thought to be a key factor in maintaining pluripotency [28]. OCT-4 is a transcription factor, which has been shown to be essential for somatic cell reprogramming, and displays various functions depending on level of expression [29,30]. Decreased expression of these factors is a positive indication of cell differentiation. 

In our project, we found that only one medium (OsteoMAX-XF™ Differentiation Medium) has shown (i) the strongest mineralization even after the short time of induction (after six days), (ii) decreased expression of specific CD multipotency markers (CD9, CD90), (iii) decreased levels in the expression of pluripotency genes (*NANOG, OCT-4*), (iv) increased expression of some specific osteogenic genes and their protein products (BSP and OCN), as well as (v) increased expression in one of the markers for differentiation (*ALP*). 

## 4. Materials and Methods 

### 4.1. Isolation of Dental Pulp Stem Cells

DPSCs were obtained from the third molar of a healthy patient during orthodontic treatment at University Hospital of Martin. The review board of the Ethical Committee of Jessenius Faculty of Medicine CU approved obtaining the human dental stem cells (approval no. EK 1821/2016). After removal, the tooth was soaked in phosphate-buffered saline (Sigma-Aldrich, St. Louis, MO, USA) with 300 U/mL penicillin and 300 µg/mL streptomycin (Biosera, Nuaille, France). Then, trephination-apical foramen-of-tooth was realized and the pulp chamber was carefully opened with special diamond burs (Busch, England). DPSCs were collected using the endodontic tool ProTaper X2 size 25 mm (Dentsply Sirona, York, PA, USA), and tissues were digested with collagenase, type IV (Thermo Fischer, Waltham, MA, USA) for 50 min with shaking. After treatment with collagenase, the cells were aspirated in basic growth medium that contained α-minimal essential medium (α-MEM, Sigma-Aldrich, St. Louis, MO, USA) supplemented with 10% fetal bovine serum embryonic stem cell qualified (Biosera, Nuaille, France), 2 mmol/L GlutaMAX (Thermo Fischer, Waltham, MA, USA), 0.2 mmol/L ascorbic acid 2-phosphate (Sigma-Aldrich, St. Louis, MO, USA), 100 U/mL penicillin and 100 µg/mL streptomycin (Biosera, Nuaille, France). DPSCs cells were grown at 37 °C under a humidified 5% CO_2_ atmosphere. The medium was changed every three days. After reaching confluence of 80%, cells were detached with TrypLE Express Enzyme (Thermo Fischer, Waltham, MA, USA), harvested and then seeded at a density of 5000 cells/cm^2^. Cells at passages no. 2–4 were used for experiments. 

### 4.2. Osteogenic Differentiation of DPSCs

We investigated the differentiation potential of DPSCs towards osteogenic lineages using various osteogenic differentiation media in order to optimize their therapeutic use. We used three different types of osteogenic differentiation media from various companies: StemPro^®^ Osteogenesis Differentiation Kit (Thermo Fischer, Waltham, MA, USA), Mesenchymal Stem Cell Osteogenic Differentiation Medium (PromoCell, Heilderberg, Germany) and OsteoMAX-XF^TM^ Differentiation Medium (Sigma-Aldrich, St. Louis, MO, USA). Osteogenic differentiation of DPSCs was induced with commercial osteogenic medium (the protocol of each producer was followed). Non-differentiated DPSCs cells (control) were cultured in basic growth medium along with differentiated cells. The osteogenic medium was changed every three days. Cell morphology was monitored by light microscopy (Optika, Ponteranica, Italy). 

### 4.3. Alizarin Red S Staining and Light Microscopy

Non-differentiated and differentiated cells were cultured on 12-well plates. After 15 days of incubation, the cells were washed with DPBS and fixed with 4% paraformaldehyde (EMS, Hatfield, PA, USA) for 30 min, in the dark, at room temperature. Fixed cells were washed twice with DPBS, and mineral deposits were stained with 2% Alizarin Red S (Merck, Darmstadt, Germany) for 3 min. After staining, cells were washed twice with water and analyzed by light microscopy (Optika, Ponteranica, Italy).

### 4.4. Flow Cytometry Analysis

Flow cytometry analysis was performed on non-differentiated and differentiated cells. Singlecell suspension was prepared by passing cells through a 70 µm strainer (Corning, New York, USA). Then, the cells were incubated in blocking buffer (1 mmol/L EDTA + 5% mouse serum in DPBS) for one hour at 4 °C, in the dark. After incubation, 5 µL of fluorescently labelled antibody, CD9-PE and CD90-APC (BioLegend, San Diego, CA, USA), were added to 0.1 × 10^6^ cells in 100 µL of buffer and incubated for 1 h at 4 °C, in the dark. Finally, the cells were centrifuged, washed with DPBS, and the cell pellet was resuspended in blocking buffer. The cells were analyzed with the FACS Aria™II cytometer (BD Biosciences, San Jose, CA, USA). The results are based on three independent experiments and data are presented as the means ± SD. Statistical analysis was performed by paired *t* test and one-way analyses of variance (ANOVA).

### 4.5. RNA Isolation and Evaluation of Gene Expression by RT-PCR

Total RNA from non-differentiated and differentiated cells was isolated using the RNeasy Mini Kit (Qiagen, Hilden, Germany). For cDNA synthesis, one microgram of total extracted RNA was applied toward cDNA generation with the RevertAid First Strand cDNA Synthesis Kit (Thermo Fischer, Waltham, MA, USA), according to the manufacturer’s instructions. PCR amplification of genes *ALP* (FW 5’GAGCTTCAGAAGCTCAACAC3’; REV 5’CTCGTTGTCTGAGTACCAGTC3’), *BSP* (FW 5’CACCACAGAGACCGGAAGGC3’; REV 5’TCCCAGGCTGGAGCTTCACT3’), *OCN* (FW 5’ACACCATGAGAGCCCTCACA3’; REV 5’AGCAGAGCGACACCCTAGAC3’), *RUNX2* (FW 5’CACTCACTACCACACCTACC3’; REV 5’TTCCATCAGCGTCAACACC3’), *NANOG* (FW 5’GGCAAACAACCCACTTCTGC3’; REV 5’CAGGACTGGATGTTCTGGGTCT3’), *OCT-4* (FW 5’CGACCATCTGCCGCTTTGAG3’; REV 5’CCCCCTGTCCCCCATTCCTA3’) and *GAPDH* (FW 5’CGGGAAGCTTGTCATCAATGG3’; REV 5’GGCAGTGATGGCATGGACTG3’) was performed with Taq DNA polymerase (Thermo Fischer, Waltham, MA, USA) following the instructions. PCR products were analyzed in 1.5% agarose gel electrophoresis (Sigma-Aldrich, St. Louis, MO, USA) at 70 V for 1 h. Bands were visualized using 1 µg/mL GelRed (Biotium, Fremont, CA, USA) on ChemiDocXRS System (BioRad, Watford, UK). The results are based on three independent experiments.

### 4.6. Immunocytochemistry and Confocal Microscopy

Immunocytochemistry was performed on both types of cells: control and osteogenic-differentiated cells. After 15 days of incubation, the cells were washed with DPBS and fixed with 4% paraformaldehyde for 30 min. Fixed cells were washed twice with DPBS and permeabilized by 0.2% Triton X-100 in DPBS (Sigma-Aldrich, St. Louis, MO, USA). After blocking with 5% goat serum (Cell Signaling Technologies, Danvesrs, MA, USA) in DBPS, stem cells and osteogenic-differentiated cells were processed for immunocytochemistry with primary rabbit antihuman BSP antibody (1:50; Cell Signaling Technologies, Danvesrs, MA, USA) or with mouse antihuman OCN antibody (1:80; Abcam, Cambridge, UK) at 4 °C, overnight. After washing, cells were treated for one hour with secondary antibodies: goat antirabbit IgG Alexa Fluor 555 (1:1000; Invitrogen, Carlsbad, CA, USA) or goat antimouse IgG Alexa Fluor 594 (1:1000; Abcam, Cambridge, UK), respectively. Cover slips with stained cells were mounted with Fluoroshield Medium supplemented with DAPI (Sigma-Aldrich, St. Louis, MO, USA). Fluorescence images were acquired using the Carl Zeiss LSM 880 NLO (Carl Zeiss AG, Jena, Germany) multiphoton confocal imaging system. In the analysis of fixed cells, W Plan Apochromat 40x/1.0 DIC objective was used. 

## 5. Conclusions

There are several kinds of adult stem cells, including bone marrow-derived mesenchymal stem cells, which are the most widely studied and utilized in clinical settings. Recently, dental pulp stem cells could be considered as potential alternatives in stem cell clinical research. This is because of their high proliferative ability, immunocompatibility and lack of ethical concerns. They can be easily, safely and almost painlessly obtained from wisdom teeth during routine dental practice. Their great differentiation potential and non-malignant phenotype can be exploited in adult stem cell research as a powerful tool in the field of regenerative medicine. However, further studies for DPSC applications in personalized therapy are needed.

## Supplementary Materials

Supplementary materials can be found at https://www.mdpi.com/1422-0067/21/7/2280/s1.

## References

[1] Dimitriou R., Jones E., McGonagle D., Giannoudis P.V. (2011). Bone regeneration: Current concepts and future directions. BMC Med..

[2] Tuček L., Kočí Z., Kárová K., Doležalová H., Suchánek J. (2017). The Osteogenic Potential of Human Nondifferentiated and Pre-differentiated Mesenchymal Stem Cells Combined with an Osteoconductive Scaffold—Early Stage Healing. Acta Medica.

[3] Anitua E., Troya M., Zalduendo M. (2018). Progress in the use of dental pulp stem cells in regenerative medicine. Cytotherapy.

[4] Karamzadeh R., Eslaminejad M.B., Aflatoonian R. (2012). Isolation, characterization and comparative differentiation of human dental pulp stem cells derived from permanent teeth by using two different methods. J. Vis. Exp..

[5] Gronthos S., Mankani M., Brahim J., Robey P.G., Shi S. (2000). Postnatal human dental pulp stem cells (DPSCs) In Vitro and In Vivo. Proc. Natl. Acad. Sci. USA.

[6] Iwamoto T., Nakamura T., Ishikawa M., Yoshizaki K., Sugimoto A., Ida-Yonemochi H., Ohshima H., Saito M., Yamada Y., Fukumoto S. (2017). Pannexin 3 regulates proliferation and differentiation of odontoblasts via its hemichannel activities. PLoS ONE.

[7] Chieruzzi M., Pagano S., Moretti S., Pinna R., Milia E., Torre L., Eramo S. (2016). Nanomaterials for Tissue Engineering in Dentistry. Nanomaterials.

[8] Jensen J., Tvedesøe C., Rölfing J.H., Foldager C.B., Lysdahl H., Kraft D.C., Chen M., Baas J., Le D.Q., Bünger C.E. (2016). Dental pulp-derived stromal cells exhibit a higher osteogenic potency than bone marrow-derived stromal cells In Vitro and in a porcine critical-size bone defect model. SICOT J..

[9] Yamada Y., Nakamura-Yamada S., Kusano K., Baba S. (2019). Clinical Potential and Current Progress of Dental Pulp Stem Cells for Various Systemic Diseases in Regenerative Medicine: A Concise Review. Int. J. Mol. Sci..

[10] Noda S., Kawashima N., Yamamoto M., Hashimoto K., Nara K., Sekiya I., Okiji T. (2019). Effect of cell culture density on dental pulp-derived mesenchymal stem cells with reference to osteogenic differentiation. Sci. Rep..

[11] Suchánek J., Soukup T., Ivancaková R., Karbanová J., Hubková V., Pytlík R., Kucerová L. (2007). Human dental pulp stem cells isolation and long-term cultivation. Acta Medica.

[12] Yasui T., Mabuchi Y., Morikawa S., Onizawa K., Akazawa C., Nakagawa T., Okano H., Matsuzaki Y. (2017). Isolation of dental pulp stem cells with high osteogenic potential. Inflamm. Regen..

[13] Ajlan S.A., Ashri N.Y., Aldahmash A.M., Alnbaheen M.S. (2015). Osteogenic differentiation of dental pulp stem cells under the influence of three different materials. BMC Oral Health..

[14] Kunimatsu R., Nakajima K., Awada T., Tsuka Y., Abe T., Ando K., Hiraki T., Kimura A., Tanimoto K. (2018). Comparative characterization of stem cells from human exfoliated deciduous teeth, dental pulp, and bone marrow-derived mesenchymal stem cells. Biochem. Biophys. Res. Commun..

[15] Polo-Corrales L., Latorre-Esteves M., Ramirez-Vick J.E. (2014). Scaffold design for bone regeneration. J. Nanosci. Nanotechnol..

[16] Verma K., Bains R., Bains V.K., Rawtiya M., Loomba K., Srivastava S.C. (2014). Therapeutic potential of dental pulp stem cells in regenerative medicine: An overview. Dent. Res. J..

[17] Potdar P.D., Jethmalani Y.D. (2015). Human dental pulp stem cells: Applications in future regenerative medicine. World J. Stem Cells.

[18] Oikonomopoulos A., van Deen W.K., Manansala A.R., Lacey P.N., Tomakili T.A., Ziman A., Hommes D.W. (2015). Optimization of human mesenchymal stem cell manufacturing: The effects of animal/xeno-free media. Sci. Rep..

[19] Cimino M., Gonçalves R.M., Barrias C.C., Martins M.C.L. (2017). Xeno-Free Strategies for Safe Human Mesenchymal Stem/Stromal Cell Expansion: Supplements and Coatings. Stem Cells Int..

[20] Castrén E., Sillat T., Oja S., Noro A., Laitinen A., Konttinen Y.T., Lehenkari P., Hukkanen M., Korhonen M. (2015). Osteogenic differentiation of mesenchymal stromal cells in two-dimensional and three-dimensional cultures without animal serum. Stem Cell Res. Ther..

[21] Egusa H., Sonoyama W., Nishimura M., Atsuta I., Akiyama K. (2012). Stem cells in dentistry—Part I: Stem cell sources. J. Prosthodont. Res..

[22] Moraes D., Sibov T.T., Pavon L.F., Alvim P.Q., Bonadio R.S., Da Silva J.R., Pic-Taylor A., Toledo O.A., Marti L.C., Azevedo R.B. (2016). A reduction in CD90 (THY-1) expression results in increased differentiation of mesenchymal stromal cells. Stem Cell Res. Ther..

[23] Maleki M., Ghanbarvand F., Reza Behvarz M., Ejtemaei M., Ghadirkhomi E. (2014). Comparison of Mesenchymal Stem Cell Markers in Multiple Human Adult Stem Cells. Int. J. Stem Cells.

[24] Lin C.S., Xin Z.C., Dai J., Lue T.F. (2013). Commonly used mesenchymal stem cell markers and tracking labels: Limitations and challenges. Histol. Histopathol..

[25] Ghaneialvar H., Soltani L., Rahmani H.R., Lotfi A.S., Soleimani M. (2018). Characterization and Classification of Mesenchymal Stem Cells in Several Species Using Surface Markers for Cell Therapy Purposes. Indian J. Clin. Biochem..

[26] Mizuno M., Kuboki Y. (2001). Osteoblast-related gene expression of bone marrow cells during the osteoblastic differentiation induced by type I collagen. J. Biochem..

[27] Liang Y., Russell I., Walworth C., Chen C. (2009). Gene expression in stem cells. Crit. Rev. Eukaryot. Gene Expr..

[28] Nakashima K., de Crombrugghe B. (2003). Transcriptional mechanisms in osteoblast differentiation and bone formation. Trends Genet..

[29] Katagiri T., Takahashi N. (2002). Regulatory mechanisms of osteoblast and osteoclast differentiation. Oral Dis..

[30] Rutkovskiy A., Stensløkken K.O., Vaage I.J. (2016). Osteoblast Differentiation at a Glance. Med. Sci. Monit. Basic Res..

