# Supplementary material for: A Comparative In Vitro Analysis of the Osteogenic Potential of Human Dental Pulp Stem Cells Using Various Differentiation Conditions
Source: Int J Mol Sci. 2020 Mar 26;21(7):2280. doi: 10.3390/ijms21072280 (PMC7177908; doi:10.3390/ijms21072280)
Supplement: Supplementary file 1 [file ijms-21-02280-s001.pdf]

Supplementary data

We performed flow cytometry analysis using positive mesenchymal lineage markers (CD44-FITC, CD90-APC, CD105-PE, CD73-PE from BioLegend, USA). and negative hematopoietic lineage marker (CD34-PE) to characterize DPSC lineage (Figure 1.). We observed similar pattern of CD marker distribution as is generally accepted:

1. Bakopoulou, A.; Leyhausen, G.; Volk, J.; Koidis, P.; Geurtsen, W. Comparative characterization of STRO-1$^{(neg)}$/CD146$^{(pos)}$ and STRO-1$^{(pos)}$/CD146$^{(pos)}$ apical papilla stem cells enriched with flow cytometry. *Arch. Oral Biol*. **2013**, 58, 1556-1568.

2. Ramos, T.L.; Sánchez-Abarca, L.I.; Muntión, S.; Preciado, S.; Puig, N.; López-Ruano, G.; Hernández-Hernández, Á.; Redondo, A.; Ortega, R.; Rodríguez, C.; Sánchez-Guijo, F.; del Cañizo, C. MSC surface markers (CD44, CD73, and CD90) can identify human MSC-derived extracellular vesicles by conventional flow cytometry. *Cell Commun. Signal*. **2016**, 14, 2.

3. Yamada, Y.; Fujimoto, A.; Ito, A.; Yoshimi, R.; Ueda, M. Cluster analysis and gene expression profiles: a cDNA microarray system-based comparison between human dental pulp stem cells (hDPSCs) and human mesenchymal stem cells (hMSCs) for tissue engineering cell therapy. *Biomaterials*. **2006** 27, 3766-3781.

4. Mokry, J.; Soukup, T.; Micuda, S.; Karbanova, J.; Visek, B.; Brcakova, E.; Suchanek, J.; Bouchal, J.; Vokurkova, D.; Ivancakova, R. Telomere attrition occurs during ex vivo expansion of human dental pulp stem cells. *J. Biomed. Biotechnol*. **2010**, 673513.

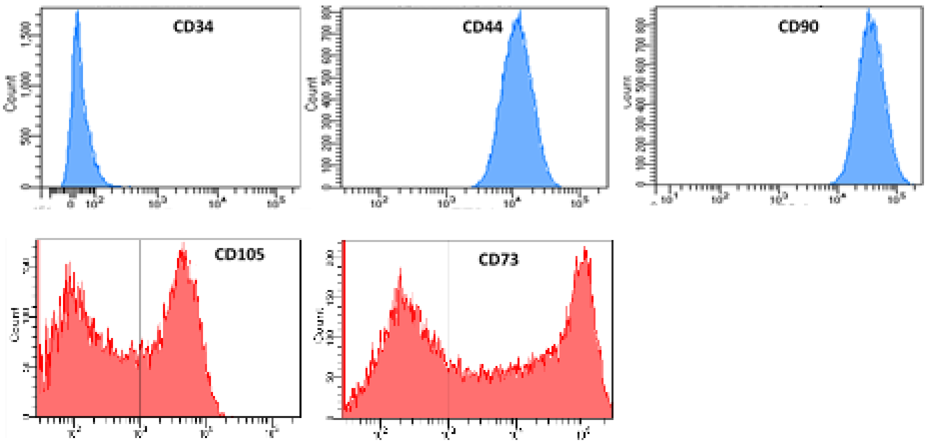

Supplementary Figure 1. Characterization of mesenchymal stem positive and negative surface markers by flow cytometry.
